# Stereotactic Body Radiotherapy for High-Risk Prostate Cancer: A Systematic Review

**DOI:** 10.3390/cancers13040759

**Published:** 2021-02-12

**Authors:** Robert Foerster, Daniel Rudolf Zwahlen, Andre Buchali, Hongjian Tang, Christina Schroeder, Paul Windisch, Erwin Vu, Sati Akbaba, Tilman Bostel, Tanja Sprave, Constantinos Zamboglou, Thomas Zilli, Jean-Jacques Stelmes, Tejshri Telkhade, Vedang Murthy

**Affiliations:** 1 Institute for Radiation Oncology, Cantonal Hospital Winterthur (KSW), 8401 Winterthur, Switzerland; daniel.zwahlen@ksw.ch (D.R.Z.); hongjian.tang@ksw.ch (H.T.); christina.schroeder@ksw.ch (C.S.); paul.windisch@ksw.ch (P.W.); 2 Medical Faculty, University of Zurich (UZH), 8091 Zurich, Switzerland; 3 Department of Radiation Oncology, Ruppiner Kliniken GmbH, Brandenburg Medical School (MHB), 16816 Neuruppin, Germany; a.buchali@ruppiner-kliniken.de; 4 Center for Proton Therapy, Paul Scherrer Institute (PSI), ETH Domain, 5232 Villingen, Switzerland; 5 Department of Radiation Oncology, Cantonal Hospital St. Gallen (KSSG), 9007 St. Gallen, Switzerland; erwin.vu@kssg.ch; 6 Department of Radiation Oncology, University Hospital Mainz, 55131 Mainz, Germany; sati.akbaba@unimedizin-mainz.de (S.A.); tilman.bostel@unimedizin-mainz.de (T.B.); 7 Department of Radiation Oncology, University Hospital Freiburg, 79106 Freiburg, Germany; tanja.sprave@uniklinik-freiburg.de (T.S.); constantinos.zamboglou@uniklinik-freiburg.de (C.Z.); 8 Department of Radiation Oncology, University Hospital Geneva (HUG), 1205 Geneva, Switzerland; thomas.zilli@hcuge.ch; 9 Department of Radiation Oncology, Oncological Institute of Southern Switzerland (IOSI), Cantonal Hospitals (EOC), 6500 Bellinzona, Switzerland; jean-jacques.stelmes@eoc.ch; 10 Department of Radiation Oncology, Tata Memorial Hospital and Advanced Centre for Treatment Research and Education in Cancer (ACTREC), Homi Bhabha National Institute (HBNI), Mumbai 400012, India; tej3003@gmail.com (T.T.); vmurthy@actrec.gov.in (V.M.)

**Keywords:** high-risk prostate cancer, stereotactic body radiotherapy, androgen deprivation therapy, toxicity, biochemical control, toxicity, review

## Abstract

**Simple Summary:**

Stereotactic body radiotherapy, i.e., high-precision radiotherapy delivering high doses within a few treatment sessions, is a very convenient treatment option, which has been shown to be effective and well tolerated in prostate cancer patients with low- or intermediate-risk profiles. This review summarizes the available data and analyzes, whether this modern treatment may routinely be offered to prostate cancer patients with a high-risk profile.

**Abstract:**

Background: Radiotherapy (RT) is an established, potentially curative treatment option for all risk constellations of localized prostate cancer (PCA). Androgen deprivation therapy (ADT) and dose-escalated RT can further improve outcome in high-risk (HR) PCA. In recent years, shorter RT schedules based on hypofractionated RT have shown equal outcome. Stereotactic body radiotherapy (SBRT) is a highly conformal RT technique enabling ultra-hypofractionation which has been shown to be safe and efficient in patients with low- and intermediate-risk PCA. There is a paucity of data on the role of SBRT in HR PCA. In particular, the need for pelvic elective nodal irradiation (ENI) needs to be addressed. Therefore, we conducted a systematic review to analyze the available data on observed toxicities, ADT prescription practice, and oncological outcome to shed more light on the value of SBRT in HR PCA. Methods: We searched the PubMed and Embase electronic databases for the terms “prostate cancer” AND “stereotactic” AND “radiotherapy” in June 2020. We adhered to the Preferred Reporting Items for Systematic Reviews and Meta-Analyses (PRISMA) recommendations. Results: After a rigorous selection process, we identified 18 individual studies meeting all selection criteria for further analyses. Five additional studies were included because their content was judged as relevant. Three trials have reported on prostate SBRT including pelvic nodes; 2 with ENI and 1 with positive pelvic nodes only. The remaining studies investigated SBRT of the prostate only. Grade 2+ acute genitourinary (GU) toxicity was between 12% and 46.7% in the studies investigating pelvic nodes irradiation and ranged from 0% to 89% in the prostate only studies. Grade 2+ chronic GU toxicity was between 7% and 60% vs. 2% and 56.7%. Acute gastrointestinal (GI) grade 2+ toxicity was between 0% to 4% and 0% to 18% for studies with and without pelvic nodes irradiation, respectively. Chronic GI grade 2+ toxicity rates were between 4% and 50.1% vs. 0% and 40%. SBRT of prostate and positive pelvic nodes only showed similar toxicity rates as SBRT for the prostate only. Among the trials that reported on ADT use, the majority of HR PCA patients underwent ADT for at least 2 months; mostly neoadjuvant and concurrent. Biochemical control rates ranged from 82% to 100% after 2 years and 56% to 100% after 3 years. Only a few studies reported longer follow-up data. Conclusion: At this point, SBRT with or without pelvic ENI cannot be considered the standard of care in HR PCA, due to missing level 1 evidence. Treatment may be offered to selected patients at specialized centers with access to high-precision RT. While concomitant ADT is the current standard of care, the necessary duration of ADT in combination with SBRT remains unclear. Ideally, all eligible patients should be enrolled in clinical trials.

## 1. Introduction

Prostate cancer (PCA) is a substantial disease burden in males with 1,276,106 new cases and 358,989 attributable deaths worldwide in 2018 [1]. Radiotherapy (RT) is an established, potentially curative treatment option for all risk constellations of localized PCA. The combination of androgen deprivation therapy (ADT) with RT has been shown to improve overall survival for patients with intermediate- and high-risk (HR) PCA [2,3,4]. Several studies have shown a superior biochemical control of dose-escalated external beam RT (EBRT) over standard-dose EBRT [5,6,7,8,9], and dose-escalation with low dose-rate (LDR) or high dose-rate (HDR) brachytherapy boost may be even superior over dose-escalated EBRT [10,11,12]. The brachytherapy boost has turned out to be an effective treatment concept because PCA seems to be more susceptible to higher single-fraction doses and a shortened overall treatment time, due to a low alpha/beta ratio [13,14]. Since dose-escalated RT traditionally requires an overall treatment time of approximately 8 weeks and BT is an invasive method with limited availability at many RT facilities, shorter and more convenient treatment schedules are desirable. In recent years, four large non-inferiority randomized trials have compared hypofractionated EBRT with standard fractionation EBRT [15,16,17,18] and, particularly, the PROFIT and CHHiP trials have shown equal outcome and comparable toxicity with hypofractionated vs. dose-escalated conventional EBRT [15,16]. Stereotactic body radiotherapy (SBRT) is a highly conformal RT technique enabling the delivery of ultra-hypofractionation, thereby substantially shortening the overall treatment time [19,20,21]. The available studies convincingly show that SBRT can be safely administered with excellent outcome in patients with low- and intermediate-risk PCA [22,23,24,25]. However, the role of SBRT in HR PCA patients remains uncertain, since only few patients were included in the conducted clinical trials [23]. Further evidence comes from several small single-arm prospective and retrospective cohorts [26,27,28,29,30,31,32,33,34,35,36,37,38,39,40,41,42,43,44,45,46,47,48,49,50,51]. One particular question that needs to be addressed, with respect to safety and efficacy of SBRT use in HR patients, is the necessity for and feasibility of elective pelvic nodal irradiation [52,53]. Therefore, we conducted this systematic review to shed more light on the value of SBRT in patients with HR PCA.

## 2. Results

### 2.1. Selected Studies

**Figure 1:**
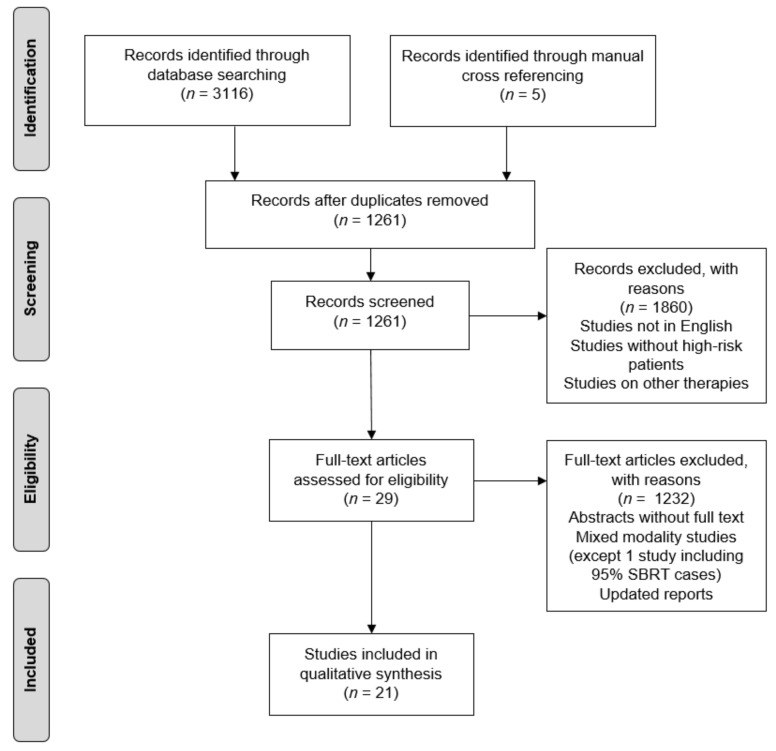
Consort diagram of the study selection process.

We identified 771 and 2345 studies searching the PubMed and Embase electronic libraries, respectively. After exclusion of duplicates, commentaries, reviews, and reports not including HR patients, 29 studies remained. Furthermore, we excluded all but one of the studies on SBRT as boost after EBRT and all of the mixed studies containing patients treated with SBRT only and SBRT as boost, since this was analyzed before [54] and no new publications were identified. A multi-institutional retrospective study was included because 95% of the patients were treated with SBRT only [35]. One recent phase II trial analyzing elective pelvic nodal irradiation [44] was excluded because it was only available in abstract form and the data content was deemed insufficient. Finally, after exclusion of repeated reports on the same cohort, 18 studies were available for further analyses. Among these, we identified 1 phase III trial [23], 1 phase II trial [24], 1 pooled analysis of phase II trials [29], 4 phase I/II trials [26,27,28,38], 4 retrospective studies based on prospectively collected data [31,45,55,56], and 7 retrospective studies [34,35,36,39,48,49,50]; overall including 651 individual patients. Five additional studies were included because of relevant information, although the patients from the respective cohorts were likely, at least in part, included in repeated reports or pooled analyses; namely 4 retrospective studies based on prospectively collected data [30,40,41,47] and 1 retrospective study [42]. All 23 selected publications were available in full text form (Table 1 and Figure 1).

### 2.2. Target Volume and Prescription Dose

#### 2.2.1. Studies with Pelvic Lymph Node Irradiation

Two Canadian phase I/II trials (FASTR and SATURN) investigated the feasibility of an elective pelvic lymph node (and seminal vesicles) irradiation with 5 × 5 Gy within a simultaneous integrated boost concept delivering 5 × 8 Gy to the prostate and proximal seminal vesicles (or prostate only) [26,28,38].

In an Indian retrospective study [31] of prospectively collected data, elective nodal irradiation was applied only in patients with node positive disease (54% of all analyzed patients). They also used a simultaneous integrated boost (SIB) concept covering the prostate and seminal vesicles as well as positive lymph nodes with 5 × 7–7.45 Gy and the electively treated pelvic lymph nodes with 5 × 5 Gy.

#### 2.2.2. Studies without Pelvic Lymph Node Irradiation

The Swedish HYPO-RT-PC trial [23] was the only identified phase III trial. Patients in the experimental arm were treated with 7 × 6.1 Gy to the prostate without the seminal vesicles, whereby roughly ¾ of patients were treated with 3D-conformal RT (3DCRT). Zilli and colleagues conducted a phase II trial investigating urethra-sparing SBRT with treatment once a week vs. every other day with a prescription dose of 5 × 7.25 Gy to the prostate ± the seminal vesicles [58]. King et al. [29] reported on pooled phase II data of a multi-institutional consortium. Median prescription dose was 5 × 7.25 Gy (range, 5 × 7–8 Gy) to the prostate only. Two phase I/II trials [27,28] were conducted in Canada (pHART8 and FASTR-2). In the pHART 8 trial, patients were treated with a SIB concept delivering 5 × 6 Gy to the seminal vesicles and 5 × 8 Gy to the prostate. Patients enrolled into the FASTR-2 trial underwent SBRT with 5 × 7 Gy to the prostate and seminal vesicles.

Bolzicco et al. and Kotecha et al. [30,45] have reported on retrospectively analyzed cohorts from prospectively collected data. While Bolzicco et al. treated the prostate and one third of the seminal vesicles with 5 × 7 Gy, Kotecha et al. irradiated the prostate including the proximal seminal vesicles with 5 × 7.25 Gy applying a SIB of 5 × 10 Gy (thereby avoiding rectum, urethra and bladder).

Further evidence is limited to 8 retrospective reports [34,35,36,39,48,49,50,55]. Most of these studies applied a 5-fraction schedule with SBRT doses between 5 × 7 to 5 × 7.5 Gy. One study from South Korea delivered SBRT to the prostate and seminal vesicles with 4 × 8–9 Gy [34].

### 2.3. Acute and Late Toxicity Rates

#### 2.3.1. Studies with Pelvic Lymph Node Irradiation

The FASTR trial [28] was prematurely terminated after completion of phase I, due to high urinary (GU) and gastrointestinal (GI) toxicity. Grade 2 acute GU toxicity was 25%, grade 2 and 3 late GU toxicity rates were at 31.25% and 6.25%, respectively. While there was no acute GI toxicity reported, late grade 2, grade 3, and even grade 4 GI toxicity was seen in 25%, 18.75% and 6.25% of patients, respectively. Similarly, in the SATURN trial [26,38] comparatively high toxicity rates were observed and reported in two separate publications. The authors reported grade 2 acute GU toxicity in 46.7% of patients, grade 2 late GU toxicity in 52–60% of patients. Acute grade 2 GI toxicity was seen in 3.3% of patients and late grade 2 GI toxicity was reported in 30–32% of patients.

In contrast to the two aforementioned Canadian trials, Murthy and colleagues reported encouraging toxicity data. Grade 2 acute GU toxicity was observed in 12%, and late grade 2 and 3 GU toxicity was reported in 4.5% and 2.5%, respectively. Acute grade 2 GI toxicity was 4% and late grade 2 GI toxicity was 4%.

**Figure 2:**
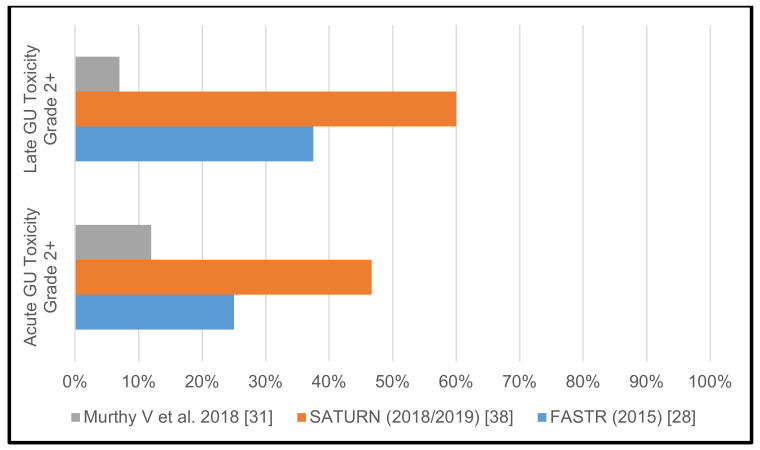
Acute and late genitourinary toxicity rates grades >/= 2 in studies with pelvic lymph node irradiation.

**Figure 3:**
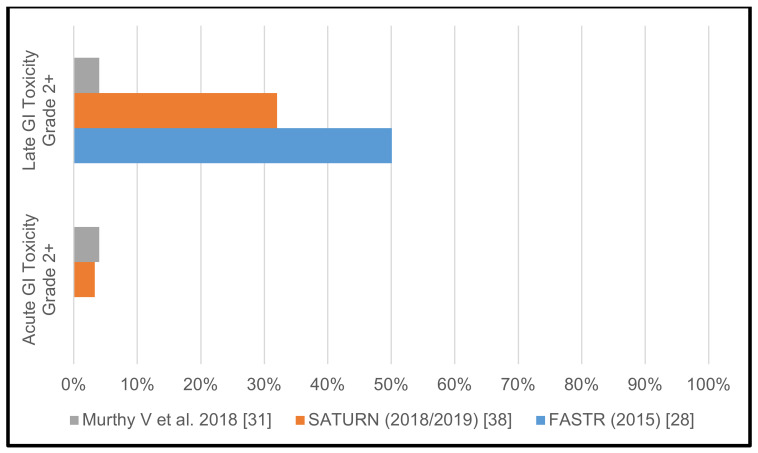
Acute and late gastrointestinal toxicity rates grades >/= 2 in studies with pelvic lymph node irradiation.

The reported >/= grade 2 GU and GI toxicity rates of the individual studies with pelvic lymph node irradiation are shown in Table 1 as well as in Figure 2 and Figure 3. Table 2 illustrates the different treatment protocols and observed toxicities of the three trials that included pelvic lymph node irradiation.

#### 2.3.2. Studies without Pelvic Lymph Node Irradiation

In their HYPO-RT-PC trial Widmark et al. [23] have included 11% HR PCA patients and conducted ultrahypofractionated RT in 62 patients. They reported that 28% of the patients in the 7 × 6.1 Gy arm experienced grade 2–4 acute GU toxicity and did not specify the acute GI toxicity rate. Grade 2–4 late GU and GI toxicity were observed in 5% and 1% of patients, respectively. Overall, they concluded that acute GU and GI toxicity was higher in the patients treated with extreme hypofractionation but late toxicity rates were similarly low in both treatment arms.

King et al. [29] did not report toxicity rates in their pooled analysis of prospective phase II trials but an individual report from the Georgetown subcohort [41] observed acute GU toxicity rates of 25% for grade 2. Acute grade 2 GI toxicity was seen in 1%. Late GU grade 2 and 3 toxicities were experienced by 17% and 1% of the patients, respectively. Grade 2 or higher GI toxicities were not observed. The Georgetown group [47] have also published their experience in men with large prostates (>50 cm^3^), and, in this particular population, toxicity rates were substantially higher. Acute grade 2 toxicity was 89% and acute grade 2 GI toxicity was 12%. Late grade 2 and 3 GU toxicities were seen in 46% and 2%. In 2% late grade 2 GI toxicities were found.

In the phase I/II trial FASTR-2 [27], acute grade 2 GU/GI toxicities were observed 14.8/3.7%. Late grade 2 GU toxicity was 21.7%. There were no grade 2 or higher GI toxicities. The pHART 8 trial [26] only reported on late GU and GI toxicity rates. GU toxicity was limited to grade 1 and 2 (36.67% and 56.67%). GI toxicity was seen in 53%, 37% and 3% for grades 1, 2 and 3, respectively.

The Italian group from Vicenza [30] reported acute GU and GI toxicities to be limited to grade 2 with 12% and 18%, respectively. Late grade 2 and 3 GU events were found in 3% and 1% of patients, respectively. Late grade 2 GI toxicities occurred in 1%, respectively. The Cleveland group [45], in their prospective database, documented acute grade 2 GU toxicity in 38% and no acute GI toxicities. Late toxicity was also limited to grade 2 GU and GI events with 2% and 8%, respectively.

Further retrospective series have reported the following toxicity rates: grade 2 acute GU: 1.4–22.6% [34,35,39,42,48,49,56], grade 3 acute GU: 0–4% [34,35,36,39,48,49,50], grade 2 acute GI: 0.4–14% [34,35,39,42,48,49,56], grade 2 late GU: 4.4–46% [34,35,36,48,49,50], grade 3 late GU: 0–4% [34,35,36,48,49,50], grade 2 late GI: 0–11.4% [34,35,36,39,48,49,50], grade 3 late GI: 0–0.9% [34,35,36,39,48,49,50]. Some reports did not specify, whether they did not see certain toxicity grades or chose to publish only specific toxicity rates.

**Figure 4:**
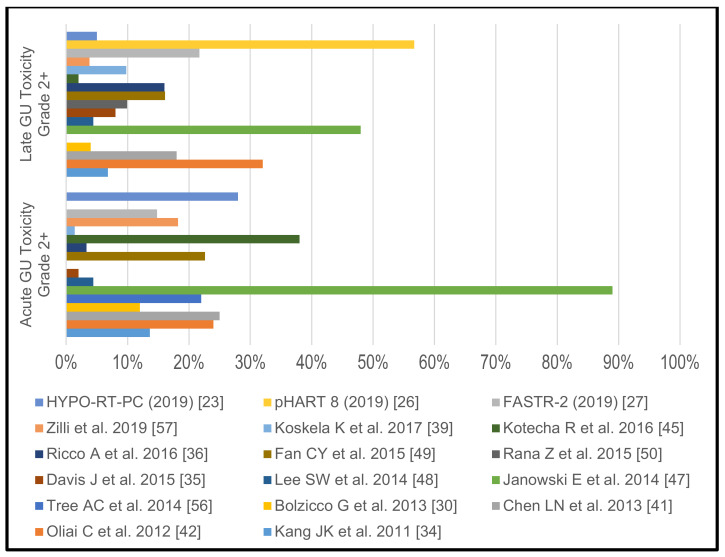
Acute and late genitourinary toxicity rates grades >/= 2 in studies without pelvic lymph node irradiation.

**Figure 5:**
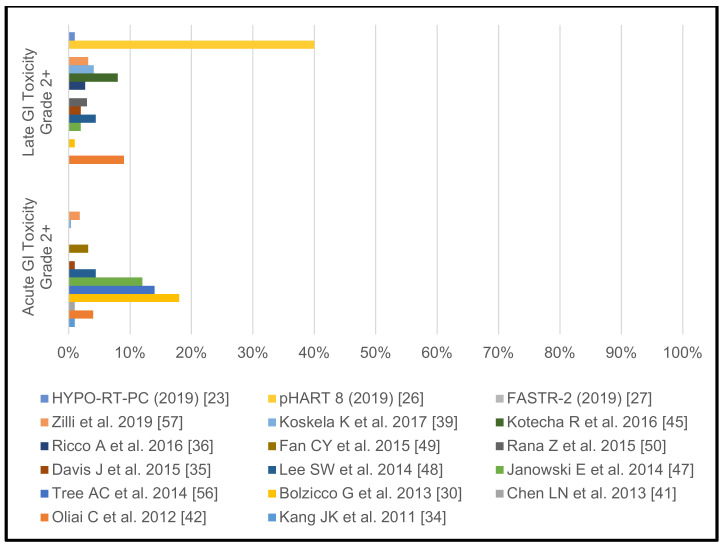
Acute and late gastrointestinal toxicity rates grades >/= 2 in studies without pelvic lymph node irradiation.

The reported >/= grade 2 GU and GI toxicity rates of the individual studies without pelvic lymph node irradiation are shown in Figure 4 and Figure 5.

### 2.4. Androgen Deprivation Therapy

#### 2.4.1. Studies with Pelvic Lymph Node Irradiation

In the FASTR trial [28], patients underwent ADT with luteinizing hormone-releasing hormone analogue (LHRHa) for 1 year. Patients in the SATURN trial [26,38] underwent ADT with LHRHa for 12–18 months.

The Mumbai cohort [31] received ADT with either LHRHa (79%) or bilateral orchiectomy (21%). LHRHa was prescribed for 2 years in node negative patients and indefinitely in those with positive lymph nodes.

#### 2.4.2. Studies without Pelvic Lymph Node Irradiation

Widmark and colleagues did not use ADT in any of their patients but only 11% of patients had HR features [23].

In the phase II trial conducted by Zilli and colleagues, a 6-month LHRHa treatment (2 months neoadjuvant) was mandatory in all patients with HR features [58]. In the US multi-institutional phase II series, 38% of the HR PCA patients underwent short-course neoadjuvant and concurrent ADT for a median duration of 4 months [29].

Both Canadian phase I/II trials prescribed neoadjuvant and concurrent LHRHa treatment for a total duration of 12 to 18 months [26,27].

Bolzicco et al. [30] did not specify how the HR PCA patients in their cohort were treated; overall, they used ADT in 29% of their patients—in 8% neoadjuvant and concurrently for 6 months and in 21% concurrently for a total median duration of 12 months. Kotecha and colleagues omitted ADT in all of their patients [45].

A Finnish retrospective study [39] used ADT in 88.3% of the HR PCA patients with 42% receiving long-term ADT for at least 2 years. Similarly, a retrospective study from Taiwan used ADT in 81% for 6 months up to 2 years in a neoadjuvant and concurrent setting [49]. In an Australian series, 50% of HR patients underwent ADT for 3–6 months [55]. In addition, a study from South Korea used ADT in their HR cohort for 2 months neoadjuvant and continued then for at least 24 months [34].

**Figure 6:**
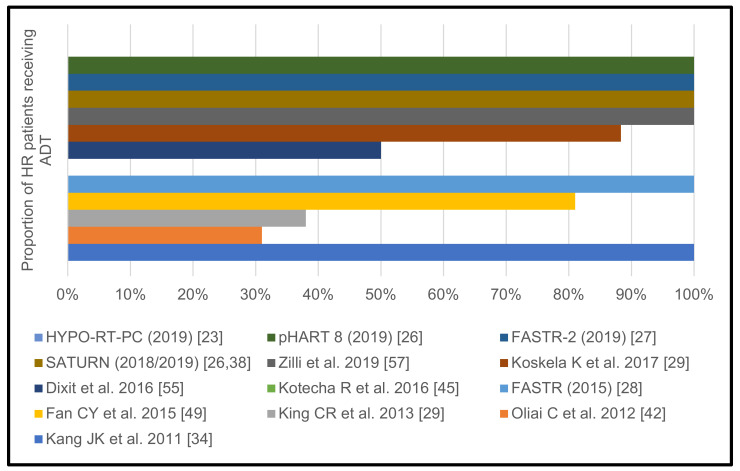
Proportion of high-risk prostate cancer patients receiving androgen deprivation therapy per individual trial.

The remaining retrospective series did not specify which percentage of their HR PCA patients underwent ADT [34,36,48,50]. ADT prescription per individual trial is shown in Figure 6.

### 2.5. Biochemical Control

#### 2.5.1. Studies with Pelvic Lymph Node Irradiation

Since the FASTR trial [28] was prematurely terminated, biochemical or biochemical progression-free survival (bPFS) rates were not reported. In the SATURN trial [26,38], biochemical control rate (BCR) was 100% after 2 years.

Patients in the Mumbai study [31] had a BCR of 94% after a median follow-up of 18 months.

#### 2.5.2. Studies without Pelvic Lymph Node Irradiation

In the HYPO-RT-PC trial [23], the 5-year disease control rate was 84% after ultrahypofractionated RT; however, they did not specify these results for the HR subgroup.

Zilli et al. did also not specify BCR of their HR patients [58]. King and colleagues reported a similar disease control probability for their HR PCA patients with a bPFS of 81.2% after 5 years in their pooled analysis of phase II data [29].

In the FASTR-2 trial [27], BCR was 100% at 12 months. In the pHART 8 trial [26], BCR was 96.6% after 2 years and 85.4% after 5 years, respectively.

Bolzicco et al. [30] and Kotecha et al. [45] reported their outcome data after shorter follow-ups. After 30 months, BCR among HR PCA patients was 94.1% in the Italian series. The colleagues from Cleveland, reported a BCR after 2 years of 84.6% for their HR patients.

The remaining retrospective analyses have reported similar disease control probabilities in HR patients after SBRT. Davis et al. [35] found a BCR among HR patients of 81.8% after a median follow-up of 20 months (range 1–64). A small UK series reported all patients to be biochemically controlled after a median follow-up of 14.5 months [56]. Rana et al. reported a BCR of 100% after 3 years [50]. A study from South Korea [34], found a BCR of 90.8% after a median follow-up of 40 months (range 12–78 months). In a study by Ricco et al. [36], after a median follow-up of 45.53 months, 87.5% of HR patients were free from biochemical failure, and in a Finish retrospective series [39], BCR was 92.8% after a median follow-up of 23 months (range 1–46 months).

Katz et al. provided the longest follow-up data for HR patients with 71% being free from failure at 7 years after SBRT [57]. More discouraging results were reported in a study by Fan and colleagues [49], where only 56.25% of HR patients were biochemically controlled after a median follow-up of 36 months (range 7–58 months).

HR-specific biochemical control rates (BCR) reported in the individual studies are shown in Table 3.

## 3. Discussion

We aimed to clarify the role of SBRT in patients with HR PCA and, in particular, to shed more light on elective pelvic nodal irradiation in combination with SBRT.

Commonly, the analyzed studies utilized a 5-fraction schedule for SBRT in HR PCA patients. Only, the HYPO-RT-PC trial and 1 older study from South Korea deviated from this approach. Most trials prescribed 5 × 7.25–7.5 Gy and there is little evidence for the safety and efficacy of further increased single-fraction doses [59,60].

As is the case for conventionally or moderately hypofractionated EBRT [14,15], some researchers chose to only irradiate the prostate and others included at least parts of the seminal vesicles. Including the seminal vesicles does not seem to lead to increased toxicity [27].

Two phase I/II trials have investigated elective pelvic lymph node RT and one large analysis of a prospective database has focused on pelvic RT in combination with prostate SBRT in the case of positive lymph nodes. While the two Canadian trials reported high toxicity rates with elective pelvic RT, the Mumbai study demonstrated the feasibility of pelvic RT. Lower toxicity in the work by Murthy et al. [27] appears to be attributable chiefly to the reduced dose prescription to the prostate (35–37.5 Gy) as compared to 40 Gy in FASTR and SATURN. Other possible reasons are a tighter posterior margin and strict planning constraints for the bladder, rectum and small bowel. The toxicity rates in the Mumbai series are in fact, closer to FASTR-2 trial despite including elective nodal irradiation to 25 Gy and treating with thrice-weekly schedule of SBRT. The retrospective nature of data collection and a different demographic profile of patients probably contributed to the differences in the toxicity rates reported. Whether elective RT of the pelvic nodes can relevantly improve outcome in the SBRT setting, will have to be addressed in more detail after the publication of the Radiation Therapy Oncology Group (RTOG) 0924 and PIVOTAL trials.

The GU and GI toxicity rates reported in the majority of the available studies merit the further investigation of SBRT in larger trials. Men with larger prostates and those irradiated with larger fields have shown to be at an increased toxicity risk [24,34,43]. The studies analyzed in this review mostly relied on robotic stereotactic radiotherapy [25,31] but state of the art conventional linear accelerator-based image-guided, intensity-modulated radiotherapy (IGRT-IMRT) without innate tracking capabilities showed convincing toxicity rates as well [19,23,27]. Whether the invasive implantation of fiducial markers can facilitate RT delivery with an improved toxicity profile remains doubtful given the high speed of current volumetric-arc therapy (VMAT) devices.

In an analogy to conventionally fractionated RT, most of the studies analyzed in this review relied on neoadjuvant and concomitant ADT prescription for patients with HR PCA and outcome being favorable. With this approach, BCR were similar to studies in intermediate-risk PCA [14,15,19]. This further is supported by the results of a recent analysis of the National Cancer Database on SBRT (*n* = 558) versus conventionally fractionated and moderately hypofractionated RT (*n* = 40,797), both combined with ADT, in men with unfavorable risk PCA, where the authors found no difference in estimated 6-year overall survival between treatment modalities [61].

Our systematic review is limited by the small number of available studies as well as by the lack of and quality of the reported data therein. A major shortcoming of our study is that we analyzed and compared prospective and retrospective studies. However, due to the paucity of prospective trials, this was unavoidable. Further limitations are the lack of an analysis of technical RT delivery data and quality of life data (QoL). Only few studies provided detailed information on contouring details, applied margins and dose constraints with a greatly variant reporting level, therefore, a useful comparison was not possible for the whole set of included studies. Similarly, the vast majority of studies did not include QoL data, therefore, we chose to limit our review to toxicity data. However, the use of toxicity scales varied greatly among the selected studies. Therefore, we compared the reported toxicity grades independently of the used toxicity scales (RTOG or Common Terminology Criteria for Adverse Events (CTC AE)). Data of some patients may have been double reported, e.g., part of the cohort from Bolzicco et al. [30] may have been included in the report by King et al. [29] and the study from Western Australia [55] stated parts of their data were published in an earlier pooled analysis. Furthermore, the definition of HR PCA was not consistent throughout the included studies, as some have chosen the D’Amico [62] and others the National Comprehensive Cancer network (NCCN) [63] classification. Given these limitations, we must conclude, that there is moderate certainty regarding the safety of SBRT to the prostate in HR PCA, but only low certainty regarding the safety of inclusion of the pelvic nodes in HR PCA. Furthermore, there is low certainty regarding the duration and timing of ADT in combination with SBRT in HR PCA as well as regarding long-term BCR after SBRT in HR PCA.

Various innovative trials are currently underway testing different fractionation schedules, positron emission tomography (PET)/magnetic resonance imaging (MRI)-guided SIB, concomitant ADT and other modern drugs in HR PCA. Until these trials are completed, further pooled prospective data analyses are strongly recommended to further clarify the role of SBRT in HR PCA patients.

## 4. Materials and Methods

### 4.1. Study Search and Selection Process

We used the PICO (population, intervention, control, outcome) criteria for the development of this review. The population was defined as men with HR PCA, the intervention was defined as primary SBRT to the pelvic nodes and/or prostate, the control was defined as primary conventionally fractionated RT to the pelvic nodes and/or prostate, and the outcome was defined as (1) rates of observed acute and chronic toxicities after SBRT to the prostate with and without pelvic nodal irradiation, (2) as rates and timing of ADT prescription with SBRT to the prostate with and without pelvic nodal irradiation, and (3) as BCR after SBRT to the prostate with and without pelvic nodal irradiation. Furthermore, we adhered to the Preferred Reporting Items for Systematic Reviews and Meta-Analyses (PRISMA) recommendations. Hence, we searched the databases PubMed and Embase for the terms “prostate cancer” AND “stereotactic” AND “radiotherapy” in June 2020. While the range of publication dates was not limited, only studies in English were eligible for selection. Abstracts mentioning the inclusion of HR cases were selected and the full texts of the respective publications were obtained and reviewed. Studies only published in abstract form were included as well. Since there are various risk classifications for PCA, we have not limited the search to any specific definition of HR PCA. After completion of the first step of the selection process, we reviewed the reference lists of all selected publications for manual cross-referencing to search for further studies to be reviewed. Studies mentioning the use of “ultra-hypofractionated radiotherapy” or “extreme fractionation” were included as well, since various technical approaches have been used in prostate cancer to deliver (highly)-conformal radiotherapy with high-doses per single fraction in a comparatively short overall treatment time [21]. Therefore, we did not exclude any specific RT delivery techniques, i.e., studies reporting on the use of robotic stereotactic radiotherapy, helical IMRT, IMRT, VMAT or 3DCRT were all eligible. Based on the authors, their affiliations, the time period, and the methods parts of the respective publications, we identified repeated reports with updated patient numbers and pooled data analyses. In case of repeated reports or pooled data analyses, the most recent publication or previous publications containing the relevant information on toxicity and/or outcome were included. To ensure a proper selection process, these tasks were independently done by two co-authors (R.F. and D.R.Z.). In the case of discrepancies, a third co-author (V.M.) reviewed the respective articles and made the final decision regarding their eligibility.

### 4.2. Data Extraction Process

We obtained the following data from the selected publications: first author, first author’s affiliation, trial name, publication year, journal, study design (prospective vs. retrospective vs. retrospective analysis of a prospective database), study period, risk classification (D’Amico vs. NCCN), number and percentages of patients included (total and HR), SBRT prescription dose, RT field (prostate only vs. prostate and pelvic lymph nodes), use of ADT (percentage of patients, duration, time point), acute and chronic toxicity rates per RTOG or CTC AE grade (I–IV), bPFS rates or BCR. To ensure proper data extraction and transcript, the data were gathered by two co-authors (R.F. and H.T.) and afterwards reviewed by another co-author (V.M.).

## 5. Conclusions

At this point, SBRT with or without pelvic ENI cannot be considered the standard of care in HR PCA, due to missing level 1 evidence. Treatment may be offered to selected patients at specialized centers with access to high-precision RT. While concomitant ADT is the current standard of care, the necessary duration of ADT in combination with SBRT remains unclear. Ideally, all eligible patients should be enrolled into clinical trials.

## Figures and Tables

**Table 1:** Overview of included studies.

Trial	Year of Publication	Type of Trial	Years Recruited	Radiotherapy (RT) Technique	Risk Classification	Number of High-Risk (HR) Patients	Prescription Dose	Androgen Deprivation Therapy (ADT) Use in HR Patients
Kang JK et al. [34]	2011	Retrospective	2002–2007	CyberKnife (CK)	D’Amico	29	4 × 8–9 Gy	100% (≥24 months, 2 months neoadjuvant)
Oliai C et al. [42]	2012	Retrospective	2007–2010	CK	D’Amico	12	5 × 7–7.5 Gy	31% (<6–24 months)
King CR et al. [29]	2013	Phase II (pooled data)	2003–2011	CK	D’Amico	121	5 × 7.25 Gy (median)	38% 4 months (median)
Bolzicco G et al. [30]	2013	Prospective database	2006–2012	CK	National Comprehensive Cancer Network (NCCN)	17	5 × 7 Gy	N/A
Chen LN et al. [41]	2013	Prospective database	2008–2010	CK	D’Amico	8	5 × 7–7.25 Gy	11% (3 weeks–36 months)
Tree AC et al. [56]	2014	Prospective database	2010–2013	CK	NCCN	6	5 × 7.25 Gy	N/A
Lee SW et al. [48]	2014	Retrospective	2006–2012	CK	NCCN	13	5 × 7.2 Gy	N/A
Janowski E et al. [47]	2014	Prospective database	2008–2011	CK	D’Amico	9	5 × 7–7.25 Gy	33.3%
Davis J et al. [35]	2015	Retrospective	2006–2015	CK Linear accelerator (Linac)	NCCN	33	5 × 7.25 Gy (87%)	45.5%
Rana Z et al. [50]	2015	Retrospective	2008–2014	CK	D’Amico	8	5 × 7.25 Gy (median)	N/A
FASTR Baumann G et al. [28]	2015	Phase I/II	2011–2017	Linac	NCCN	16	5 × 8 Gy (prostate) and 5 × 5 Gy (pelvic elective nodal irradiation (ENI))	100% (12 months)
Fan CY et al. [49]	2015	Retrospective	2010–2013	CK	NCCN	16	5 × 7.5 Gy	81% (6–24 months, neoadjuvant)
Dixit A et al. [55]	2016	Prospective database	2014–2015	CK	D’Amico	6	5 × 7.25 Gy	50% (3–6 months)
Kotecha R et al. [45]	2016	Prospective database	2011–2014	Linac	NCCN	13	5 × 7.25/10 Gy (simultaneous-integrated boost (SIB))	No
Ricco A et al. [36]	2016	Retrospective	2007–2012	CK	NCCN	32	5 × 7–7.25 Gy	N/A
Katz A et al. [57]	2016	Prospective database	2006–2010	CK	NCCN	38	5 × 7–7.25 Gy	55.3% (6 months, neoadjuvant)
Koskela K et al. [39]	2017	Retrospective	2012–2015	CK	D’Amico	111	5 × 7–7.25 Gy	88.3% (48% for ≥2 years)
Murthy V et al. [31]	2018	Prospective database	2014–2017	Tomotherapy Linac	NCCN	68	5 × 7–7.45 Gy (prostate) and 5 × 5 Gy (cN1)	100% (≥2 years)
SATURN Alayed Y et al. [26] Musunuru HB et al. [38]	2018/2019	Phase I/II	2013–2014	Linac	NCCN	30	5 × 8 Gy (prostate) and 5 × 5 Gy (pelvic ENI)	100% (12–18 months)
HYPO-RT-PC Widmark A et al. [23]	2019	Phase III	2005–2015	Linac	NCCN	62	7 × 6.1 Gy	No
FASTR-2 Callan L et al. [27]	2019	Phase I/II	2015–2017	Linac	NCCN	28	5 × 7 Gy	100% (18 months, 2 months neoadjuvant)
pHART8 Alayed Y et al. [26]	2019	Phase I/II	2011–2013	Linac	NCCN	30	5 × 6/8 Gy (SIB)	100% (12–18 months)
Zilli T et al. [58]	2020	Phase II	2012–2015	Linac	NCCN	29	5 × 7.25 Gy	100% (6 months, 2 months neoadjuvant)

**Table 2:** Treatment characteristics and observed toxicities of the three trials that included pelvic lymph node irradiation.

Parameters	FASTR [28]	SATURN [38]	Murthy et al. 2018 [31]	
Primary clinical target volume (CTV)	Prostate + 1 cm seminal vesicles (SV)	Prostate	Prostate + entire SV	
Primary CTV to planning target volume (PTV)	5 mm	3 mm	5 mm (3 mm posteriorly)	
Dose to prostate	40 Gy to PTV	40 Gy to CTV 33.25 Gy to PTV	35 Gy–37.5 Gy to PTV	
Pelvic lymph node irradiation	25 Gy to PTV	25 Gy to CTV 23.75 Gy to PTV	25 Gy to PTV	
Fractionation	Once weekly	Once weekly	Thrice weekly	
Image guidance	Cone beam computed tomography (CBCT)	CBCT + fiducials	CBCT	
Bladder dose constraints	V35 < 30% V29 < 50%	V35 < 5% V32 < 10%	V35 < 3% V17.5 < 20%	
Rectum dose constraints	V35 < 20% V27 < 50%	V35 < 5% V32 < 10%	V35 < 3% V31.5 < 8% V28 < 15% V17.5 < 40%	
Small bowel dose constraints	V25 < 190 cc V27.5 < 2 cc	V25 < 20 cc V30 < 2 cc	V28 < 80 cc	
Median Follow-up	6 months	24 months	18 months	
Grade 2+ acute gastrointestinal (GI) toxicity	0.0%	3.3%	4.0%	
Grade 2+ acute genitourinary (GU) toxicity	25%	46.7%	12.0%	
Grade 2+ late GI toxicity	50.1%	32.0%	4.0%	
Grade 2+ late GU toxicity	37.5%	60.0%	7.0%	

**Table 3:** Biochemical control rates reported in the individual studies.

Studies	~1 year	~2 years	~3 years	~4 years	5 years	7 years
Kang JK et al., 2011 [34]	-	-	91%	-	-	-
King CR et al., 2013 [29]	-	-	-	-	81%	-
Bolzicco G et al., 2013 [30]	-	-	94%	-	-	-
Tree AC et al., 2014 [56]	100%	-	-	-	-	-
Davis J et al., 2015 [35]	-	82%	-	-	-	-
Rana Z et al., 2015 [50]	-	-	100%	-	-	-
Fan CY et al., 2015 [49]	-	-	56%	-	-	-
Ricco A et al., 2016 [36]	-	-	-	88%	-	-
Kotecha R et al., 2016 [45]	-	85%	-	-	-	-
Katz A et al., 2016 [57]	-	-	-	-	-	71%
Koskela K et al., 2017 [39]	-	93%	-	-	-	-
Murthy V et al., 2018 [31]	-	94%	-	-	-	-
SATURN (2018/2019) [38]	-	100%	-	-	-	-
FASTR-2 (2019) [27]	100%	-	-	-	-	-
pHART 8 (2019) [26]	-	97%	-	-	85%	-

## Data Availability

All data are available by request from the corresponding author.
